# Prevalence and Associated Risk Factors of Gestational Diabetes Mellitus at a University Hospital in Saudi Arabia

**DOI:** 10.12669/pjms.35.2.498

**Published:** 2019

**Authors:** Shahad Abualhamael, Hala Mosli, Mukhtiar Baig, Abdulelah M. Noor, Fahd M. Alshehri

**Affiliations:** 1 *Dr. Shahad Abualhamael, SBIM, Department of Medicine, Faculty of Medicine, King Abdulaziz University, Jeddah, Saudi Arabia*; 2 *Dr. Hala Mosli, ABEM, Department of Medicine, Faculty of Medicine, King Abdulaziz University, Jeddah, Saudi Arabia*; 3 *Prof. Mukhtiar Baig, Ph.D, Department of Clinical Biochemistry, Faculty of Medicine, King Abdulaziz University, Jeddah, Saudi Arabia*; 4 *Dr. Abdulelah M. Noor, MBBS, Department of Medicine, King Abdullah Medical City, Makkah, Saudi Arabia*; 5 *Dr. Fahd M. Alshehri, MBBS, Department of Medicine, Faculty of Medicine, King Abdulaziz University, Jeddah, Saudi Arabia*

**Keywords:** BMI, GDM, Parity, Prevalence, Risk factors

## Abstract

**Objectives:**

We aimed to find out the prevalence and associated risk factors of GDM among females who attended antenatal clinic at King Abdulaziz University Hospital (KAUH), Jeddah, Saudi Arabia (SA).

**Methods:**

This retrospective study was carried out from 25^th^ September 2016 till 20^th^ December 2016, at the Endocrine Clinic, Faculty of Medicine, KAUH, Jeddah, SA. A total of 5000 women attended antenatal clinic and 637 women were referred to the endocrine clinic for GDM. The data of only 103 GDM was included in the study because only these patients’ complete data was available. The electronic record of 93 pregnant age and BMI matched females, not having GDM were selected as a control group.

**Results:**

The prevalence of GDM was 12.75% (637/5000). Parity was associated with GDM (X^2^=16.82, P=.001) and GDM was significantly higher in multigravida while no association of GDM was found with working status, place of living, hypertension, family history of DM and BMI. Logistic regression analysis revealed that grand multigravida female had the lower risk of GDM as compared to multi, primi and nulligravida while age, working status, place of living, hypertension, family history of DM and BMI were not found significant risk factors for GDM. In GDM group, according to nationality, 68(66%) women were Saudi while 35(34%) were expatriates’ (Yemeni 11.2%, Egyptians 3.9%, Indians 3.9%, Pakistanis 2.9%, Sudanese 2.9%, Syrians 2.9% and others).

**Conclusions:**

The prevalence of GDM was 12.75% and it was not associated with working status, place of living, hypertension, family history of diabetes and BMI.

## INTRODUCTION

Gestational Diabetes Mellitus (GDM) is defined as glucose intolerance that can be identified during pregnancy, usually, happening following the 24th week of gestation.^1^ It is associated with grave consequences not only for the pregnant females but also for the fetus and after delivery to the neonates.

It is important to recognize and treat the problem in early stages because GDM associated complications in mother and fetus are mostly preventable.^2^ One of the reasons for the increase in GDM prevalence is that it does not have very obvious symptoms; however, excessive urination and fatigue, urinary tract infections (UTIs), nausea and vomiting are likely to be present.^3^ If it is not diagnosed and treated then gestational hyperglycemia may cause various complications to the woman such as abortion, pre-eclampsia, preterm labor, placenta praevia, vaginal itching, UTI, puerperal sepsis, and pyelonephritis.^4^

The pattern of prevalence of GDM has notable regional and ethnic differences. Asian inhabitants are considered at higher risk than white populations.^5^ Increasing maternal age and obesity, earlier pregnancy GDM, and family history of DM are considered few dominant risk factors for the progress of GDM.^6^,^7^

The prevalence of GDM ranges from 8 to 19% in Saudi Arabia.^8^,^9^ However, a large-scale study in Riyadh (capital of SA) reported that SA has the highest prevalence of GDM (24%) in the world.^10^ In the present study, we aimed to find out the prevalence and associated risk factors of GDM among females attended antenatal clinic during the year 2015, at King Abdulaziz University Hospital (KAUH), Jeddah, SA.

## METHODS

This retrospective study was carried out from 25^th^ September 2016 till 20^th^ December 2016, at the Endocrine Clinic, Faculty of Medicine, KAUH, Jeddah, SA. One-year data of the GDM subjects were collected from their electronic records from 1^st^ January 2015 to 31^st^ December 2015. A total of 5000 women attended the antenatal clinic, and 637 women were referred to the Endocrine Clinic because of GDM. So the prevalence of GDM was 12.75% (637/5000). We collected GDM subjects’ data from the electronic record; however, the data of only 103 GDM was taken because these patients’ complete data according to our questionnaire was available. The electronic record of 93 pregnant age and BMI matched females, not having GDM were selected as a control group.

With their record, a questionnaire was filled regarding their age, BMI, blood pressure, living place, working status, family history of diabetes, and parity. We included only those subjects who were diagnosed with GDM and did not have DM simultaneously.

The American Diabetes Association (ADA) cut-off values using the one-step approach were used to classify the study subjects as GDM^11^: “Gestational diabetes mellitus should be diagnosed at any time in pregnancy if one of the following criteria are met or exceeded:

1. Fasting plasma glucose> or equal 5.1 mmol (92mg/ dl).
2. 1-Hour plasma glucose ≥ 10.0 mmol/l (180mg/ dl) following a 75 g oral glucose load.
3. 2-Hour plasma glucose 8.5 mmol/l (153 mg/dl) following a 75 g oral glucose load.”

Two or more criteria must be met or exceeded for a positive diagnosis. We excluded the diagnosed DM patients and having any other endocrinology problems such as thyroid disorders, Polycystic ovary syndrome, Cushing syndrome that affect blood sugar level. All the participants were screened during 24–28 weeks of gestation or before if they were at high risk for developing GDM. The patients were considered high risk if they had BMI greater than 30 Kg/m^2^, prior history of gestational diabetes, earlier large baby weighing 4.5 Kg or more, and family history of diabetes. The present study was approved by the ethical committee of the KAUH, Jeddah, SA. We kept the confidentiality of the subject and did not disclose any data relating to their identification.

The data were analyzed by SPSS (Statistical Package for Social Sciences) version 23. Mean ± SD was given for quantitative variables. Frequency and percentages were given for qualitative variables. Student t-test was used to compare the mean of age, HBA1C and fasting blood sugar between cases and controls. Pearson Chi-square test was used to determine the relationship of age, parity, working status, place of living, history of hypertension and BMI. Logistic regression was employed to compute the unadjusted and adjusted odds ratio by using age, parity, working status, place of living, history of hypertension, diabetes, and BMI as independent variables. The p-value < 0.05 was taken as significant.

## RESULTS

In our study, the prevalence of GDM was 12.75% (637/5000). We compared the data of 103 GDM, and 93 age and BMI matched control subjects. Our results show that HBA1C and fasting plasma glucose (FPG) were significantly higher in cases as compared to control (P<0.001) while there was no significant difference of age (P=0.14) and BMI (P=0.13) between both groups. In GDM group, 60 (58.3%) women were obese (BMI>30) while in the control group there were 45 (48.4%) had BMI greater than 30 (Table-I). The nationality-wise distribution of GDM patients is shown in Table-II.

**Table-I:** Comparison of quantitative variable of Gestational Diabetes Mellitus (GDM) patients and control group.

Variables	GDM Mean±SD (N=103)	ControlsMean±SD (N=93)	p-value
Age (Years)	33.4 ± 5.9	32.3 ± 6.1	0.14
BMI (Kg/m^2^)	31.9 ± 6.3	30.7 ± 5.1	0.13
HBA_1C_ (%)	6.25 ± 1.04	4.40 ± 0.80	< 0.001 *
FPG (mmol/L)	5.75 ± 1.08	4.29 ± 0.48	< 0.001 *

Values are given as mean ± SD, SD: Standard Deviation, BMI: Body Mass Index, FPG: Fasting Plasma Glucose, p-value is generated by Student t- test

* p-value ≤ 0.05 is considered statistically significant.

**Table-II:** Nationality-wise distribution of GDM patients.

Nationality	N	%
Saudi	68	66
Yemni	12	11.2
Egyptians	4	3.9
Indians	4	3.9
Pakistani	3	2.9
Sudanese	3	2.9
Syrians	3	2.9
Others	6	6

N: Number, %: Percentage

Parity was associated with GDM (X^2^=16.82, P=0.001) and GDM was significantly higher in multigravida while no association of GDM was found with working status, place of living, hypertension, family history of DM and BMI (Table-III).

**Table-III:** Comparison of few basic characteristics of Gestational Diabetes Mellitus (GDM) patients and control group.

Variables	GDM (N=103) N (%)	Controls^a^(N=93) N (%)	p-value
***Age Group***			
< 30 years	24 (23.3)	32 (34.4)	0.09
≥ 30 years	79 (76.7)	61 (65.65)	
***Parity***			
Nulligravida	11 (10.7)	6 (6.5)	0.001 *
Primigravida	29 (28.25)	14 (15.15)	
Multigravida	52 (50.5)	42 (45.2)	
Grand multigravida	11 (10.7)	31 (33.35)	
***Working status***			
House wife	68 (66.0)	64 (68.8)	0.68
Job	35 (34.0)	29 (31.25)	
***Residence***			
Rural	8 (7.8)	8 (8.6)	0.83
Urban	95 (92.2)	85 (91.4)	
***Hypertension***			
Yes	39 (37.9)	33 (35.5)	0.73
No	64 (62.1)	60 (64.5)	
***Family history of Diabetes***			
Yes	44 (42.7)	30 (32.3)	0.14
No	59 (57.3)	63 (67.7)	
***BMI***			
< 30	43 (41.7)	48 (51.6)	0.17
≥ 30	60 (58.35)	45 (48.4)	

N: Number, %: Percentage, BMI: body mass index, p-value is generated by Pearson Chi-Square test,

* p-value ≤ 0.05 is considered statistically significant.

Logistic regression analysis revealed that grand multigravida female had the lower risk of GDM as compared to multi, primi and nulligravida [OR=0.16(95% CI 0.05-0.56; P=0.004)] while increased age (≥ 30 years), working status, place of living, hypertension, family history of DM and BMI were not found significant risk factors for GDM (Table-IV).

**Table-IV:** Regression analysis of different variables in Gestational Diabetes Mellitus (GDM).

Variables	Odds ratio (95% Confidence interval)	p-value	Adjusted odds ratio (95% Confidence interval)*^a^*	p-value
***Age Group***				
< 30 years	1	0.08	1	0.07
≥ 30 years	1.73 (0.92 – 3.23)		1.89 (0.94 – 3.79)	
***Parity***				
Nulligravida	1		1	
Primigravida	1.13 (0.35 – 3.68)	0.84	1.04 (0.30 – 3.53)	0.95
Multigravida	0.68 (0.23 – 1.98)	0.47	0.56 (0.18 – 1.73)	0.33
Grand multigravida	0.19 (0.58 – 0.65)	0.008	0.16 (0.05 – 0.56)	0.004
***Working status***				
House wife	1	0.68	1	0.54
Job	1.14 (0.62 – 2.07)		1.22 (0.64 – 2.33)	
***Residence***				
Rural	1	0.83	1	0.81
Urban	1.12 (0.40 – 3.12)		1.14 (0.39 – 3.38)	
***Hypertension***				
Yes	1	0.73	1	0.61
No	1.12 (0.62 – 1.98)		0.84 (0.44 – 1.63)	
***Family history of Diabetes***				
Yes	1	0.13	1	0.24
No	1.57 (0.87 – 2.81)		1.46 (0.79 – 2.74)	
***BMI***				
< 30	1	0.17	1	0.34
≥ 30	1.49 (0.85 – 2.62)		1.36 (0.72 – 2.57)	

## DISCUSSION

The prevalence of GDM in our study is 12.75%, in comparison to several other studies published from SA, our results are similar to Serehi AA et al., (2015)^12^ and Al-Rowaily & Abolfotouh (2010)^13^, lower than Wahabi HA et al., (2013)^9^ and Wahabi H et al., (2017)^10^ and higher than (Abdelmola, 2017).^8^ Globally, variability is found in the prevalence of GDM, Denmark 1.7-2.9%, USA 3.9% to 12.8%, Qatar 16.3%, Pakistan 17.2%, Zimbabwe 6%, and Australia 13.2%.^6^,^14^-^18^ This difference could be due to the difference in race, genetic predisposition, dietary and lifestyle patterns, and variability in diagnostic criteria.

Logistic regression analysis revealed that age ≥ 30 years increases the risk of GDM, but it was not significant. Our results are similar to a study.^17^ In contrast to our results, Abdelmola AO et al., (2017) showed that the women in the age group (31–35) years had the significantly higher prevalence of GDM.^8^ Several other studies reported the higher prevalence of GDM among women in the age group > 35 years.^13^,^15^ This difference could be due to small sample size and variability in diagnostic criteria.

The increased age (≥30 years), working status, place of living, hypertension, family history of DM and BMI were not found significant risk factors for GDM. Similar to our results a study reported no significant association between GDM and working status.^10^ Our results are similar to a South African and a Thai study that did not find the association between obesity and GDM.^19^,^20^ However, our results are inconsistent with several other studies that have reported increased age. BMI, and multiparity as risk factors for GDM.^21^-^23^ This difference could be due to different race and use of the different criteria for obesity.

We found that grand multigravida have a lower risk for GDM after adjusting age, working status, place of living, hypertension, family history of DM and BMI. This observation is also in accordance with our result that increasing age is not a risk factor for GDM. Recently, Nhidza G et al., (2018) demonstrated that gravida is not a predictor of the GDM.^17^ Contrary to our results, Collier A et al., (2017)^21^ reported the association of GDM with multiparity while another report from Pakistan demonstrated that with increased BMI, age, and history of T2DM in the family was associated with GDM.^16^ So conflicting results have been reported in the literature. Usually, with increasing age and after several pregnancies there is increased in body weight that could be responsible for impaired OGT and GDM. However, in our study because of the small sample size this effect could not be found.

A recent study reported that the women suffering from GDM had increased chances of having a macrosomic baby without having the increased risk for other maternal or neonatal complications.^10^ Additionally, females with GDM are more prone to develop T2DM in their later lives.^24^

In GDM group, according to nationality, 68(66%) women were Saudi while 35(34%) were expatriates’ (Yemeni 11.2%, Egyptians 3.9%, Indians 3.9%, Pakistanis 2.9%, Sudanese 2.9%, Syrians 2.9% and others). The Jeddah city is the second largest and thickly populated cosmopolitan city, and thousands of expatriates’ families reside in the city. Therefore, our data comprised of women of several nationalities, so our results do not reflect the frequency of GDM among Saudi women.

It is suggested that changes in dietary pattern and sedentary lifestyle causing obesity and the more prolonged period of education and better access to birth control techniques causing increasing age at first pregnancy.^25^ These factors are likely to be involved in the widespread prevalence of GDM.

There is a need to educate the women regarding the drastic effects of the GDM for them, and for their fetus. Such education should be started at school and college level. Research indicates that prevalence of GDM is directly related to the prevalence of T2DM in the society^26^ and T2DM is highly prevalent in Saudi Arabia.^27^ Obesity is considered one of the very important reasons for T2DM because it causes resistance to insulin and consequently T2DM occurs. Therefore, we should educate the population about the healthy lifestyle and make them aware of the grave consequences of the obesity. Moreover, Riaz RH et al., (2018) suggested that the physicians’ awareness regarding GDM up to date diagnostic and management strategies could play a pivotal role in managing GDM burden.^28^

### Limitations of study

First, it has been conducted only in one hospital; consequently the sample size was small. Secondly, the data was collected from the electronic record of the patients, so, little important information could not be collected such as the history of their dietary, physical activities, socioeconomic status, educational level and others.

## CONCLUSION

Our results indicate that the prevalence of GDM was 12.75% in our setup and no association was found with working status, place of living, hypertension, family history of diabetes and BMI.

### Author`s Contribution

**SA & HM:** Contributed to conception and design of the work, acquisition of data and final approval of the version to be published.

**MB:** Contributed to interpretation of data, manuscript writing and revised the manuscript for intellectual content.

**AMN & FMA:** Contributed to data collection, analysis and write up.

## References

[1] Panel IC (2010). International association of diabetes and pregnancy study groups recommendations on the diagnosis and classification of hyperglycemia in pregnancy. Diabetes Care.

[2] Dabelea D, Snell-Bergeon JK, Hartsfield CL, Bischoff KJ, Hamman RF, McDuffie RS (2005). Increasing prevalence of gestational diabetes mellitus (GDM) over time and by birth cohort:Kaiser Permanente of Colorado GDM Screening Program. Diabetes Care.

[3] Lawal M (2008). Management of diabetes mellitus in clinical practice. Br J Nurs.

[4] Moy FM, Ray A, Buckley BS (2014). Techniques of monitoring blood glucose during pregnancy for women with pre-existing diabetes. Cochrane Database Syst Rev.

[5] Hedderson M, Ehrlich S, Sridha S, Darbinian J, Moore S, Ferrara A (2012). Racial/ethnic dis- parities in the prevalence of gestational diabetes mellitus by BMI. Diabetes Care.

[6] Jeppesen C, Maindal HT, Kristensen JK, Ovesen PG, Witte DR (2017). National study of the prevalence of gestational diabetes mellitus among Danish women from 2004 to 2012. Scand J Public Health.

[7] Hod M, Kapur A, Sacks DA, Hadar E, Agarwal M, Di Renzo GC (2015). The International Federation of Gynecology and Obstetrics (FIGO) initiative on gestational diabetes mellitus:A pragmatic guide for diagnosis, management, and care. Int J Gynecol Obst.

[8] Abdelmola AO, Mahfouz MS, Gahtani MA, Mouharrq YJ, Hakami BH, Daak OI (2017). Gestational diabetes prevalence and risk factors among pregnant women—Jazan Region, Saudi Arabia. Clin Diabetol.

[9] Wahabi HA, Esmaeil SA, Fayed A, Alzeidan RA (2013). Gestational diabetes mellitus:maternal and perinatal outcomes in King Khalid University Hospital, Saudi Arabia. J Egypt Public Health Assoc.

[10] Wahabi H, Fayed A, Esmaeil S, Mamdouh H, Kotb R (2017). Prevalence and complications of pregestational and gestational diabetes in Saudi women:analysis from Riyadh Mother and Baby cohort study (RAHMA). Bio Med Res Int.

[11] American Diabetes Association (2010). Diagnosis and classification of diabetes mellitus. Diabetes Care.

[12] Serehi AA, Ahmed AM, Shakeel F, Alkhatani K, El-Bakri NK, Buhari BA (2015). A comparison on the prevalence and outcomes of gestational versus type 2 diabetes mellitus in 1718 Saudi pregnancies. Int J Clin Exp Med.

[13] Al-Rowaily MA, Abolfotouh MA (2010). Predictors of gestational diabetes mellitus in a high-parity community in Saudi Arabia. East Mediterr Health J.

[14] Hunt KJ, Schuller KL (2007). The increasing prevalence of diabetes in pregnancy. Obstet Gynecol Clin North Am.

[15] Bener A, Saleh NM, Al-Hamaq A (2011). Prevalence of gestational diabetes and associated maternal and neonatal complications in a fast-developing community:global comparisons. Int J Womens Health.

[16] Fatima SS, Rehman R, Alam F, Madhani S, Chaudhry B, Khan TA (2017). Gestational diabetes mellitus and the predisposing factors. JPMA.

[17] Nhidza G, Mutsaka K, Malunga G, Zhou DT (2018). Diagnosis of Gestational Diabetes Mellitus in Urban Harare, Zimbabwe. Open Public Health J.

[18] McDonald R, Karahalios A, Le T, Said J (2015). A retrospective analysis of the relationship between ethnicity, body mass index, and the diagnosis of gestational diabetes in women attending an Australian antenatal clinic. Int J Endocrinol.

[19] Kongubol A, Phupong V (2011). Prepregnancy obesity and the risk of gestational diabetes mellitus. BMC Pregnancy Childbirth.

[20] Basu JK, Jeketera CM, Basu D (2010). Obesity and its outcomes among pregnant South African women. Int J Gynaecol Obstet.

[21] Collier A, Abraham EC, Armstrong J, Godwin J, Monteath K, Lindsay R (2017). Reported prevalence of gestational diabetes in Scotland:The relationship with obesity, age, socioeconomic status, smoking and macrosomia, and how many are we missing?. J Diabetes Investig.

[22] Majeed A, El-Sayed AA, Khoja T, Alshamsan R, Millett C, Rawaf S (2014). Diabetes in the Middle-East and North Africa:an update. Diabetes Res Clin Pract.

[23] Kerrigan AM, Kingdon C (2010). Maternal obesity and pregnancy:a retrospective study. Midwifery.

[24] Kim SY, England JL, Sharma JA, Njoroge T (2011). Gestational diabetes mellitus and risk of childhood overweight and obesity in offspring:A systematic review. Exp Diabetes Res.

[25] Hoem J, Neyer G, Andersson G (2006). Educational attainment and ultimate fertility among Swedish women born in 1955-59. Demogr Res.

[26] Ben-Haroush A, Yogev Y, Hod M (2004). Epidemiology of gestational diabetes mellitus and its association with Type 2 diabetes. Diabetic Med.

[27] Al-Rubeaan K, Al-Manaa HA, Khoja TA, Ahmad NA, Al-Sharqawi AH, Siddiqui K (2015). Epidemiology of abnormal glucose metabolism in a country facing its epidemic:SAUDI-DM study. J Diabetes.

[28] Riaz SH, Khan MS, Jawa A, Hassan M, Akram J (2018). Lack of uniformity in screening, diagnosis and management of gestational diabetes mellitus among health practitioners across major cities of Pakistan. Pak J Med Sci.

